# The Kinesin‐3 motor, KLP‐4, mediates axonal organization and cholinergic signaling in *Caenorhabditis elegans*

**DOI:** 10.1096/fba.2019-00019

**Published:** 2019-06-11

**Authors:** Margaret E. Magaletta, Kendall J. Perkins, Catherine P. Deuchler, Jay N. Pieczynski

**Affiliations:** ^1^ Department of Biology Rollins College Winter Park Florida; ^2^ Program in Molecular Medicine, Diabetes Center of Excellence University of Massachusetts Medical School Worcester Massachusetts

**Keywords:** axon, behavior, cholinergic signaling, kinesin, synaptic vesicle

## Abstract

Microtubule plus‐end directed trafficking is dominated by kinesin motors, yet kinesins differ in terms of cargo identity, movement rate, and distance travelled. Functional diversity of kinesins is especially apparent in polarized neurons, where long distance trafficking is required for efficient signal transduction‐behavioral response paradigms. The Kinesin‐3 superfamily are expressed in neurons and are hypothesized to have significant roles in neuronal signal transduction due to their high processivity. Although much is known about Kinesin‐3 motors mechanistically in vitro*,* there is little known about their mechanisms in vivo. Here, we analyzed KLP‐4, the *Caenorhabditis elegans* homologue of human KIF13A and KIF13B. Like other Kinesin‐3 superfamily motors, *klp‐4* is highly expressed in the ventral nerve cord command interneurons of the animal, suggesting it might have a role in controlling movement of the animal. We characterized an allele of *klp‐4* that contains are large indel in the cargo binding domain of the motor, however, the gene still appears to be expressed. Behavioral analysis demonstrated that *klp‐4* mutants have defects in locomotive signaling, but not the strikingly uncoordinated movements such as those found in *unc‐104*/KIF1A mutants. Animals with this large deletion are hypersensitive to the acetylcholinesterase inhibitor aldicarb but are unaffected by exogenous serotonin. Interestingly, this large *klp‐4* indel does not affect gross neuronal development but does lead to aggregation and disorganization of RAB‐3 at synapses. Taken together, these data suggest a role for KLP‐4 in modulation of cholinergic signaling in vivo and shed light on possible in vivo mechanisms of Kinesin‐3 motor regulation.

**Abbreviations**
AchE: acetylcholinesterase
CBD: cargo binding domain
CC: coiled‐coil
CSS: core synapse stability
FHA: forkhead associated domain
MD: motor domain
NC: neck coil
NL: neck linker
SV(s): synaptic vesicle(s)
VNC: ventral nerve cord

## 1 INTRODUCTION

The dynein and kinesin protein families are ATP dependent molecular motors that move along polarized microtubules in virtually all cell types. Both groups of motors traffic various cargoes within cells, including organelles, and vesicles. Kinesins are by far the most diverse group of motors, consisting of approximately 50 distinct proteins subdivided into 15 distinct superfamilies plus an uncategorized orphan group.^1^, ^2^ Various criteria have been used to group kinesins including, but not limited to: domain structure, direction of movement, and location of dual functioning catalytic and microtubule binding motor domains. Although slight differences exist in kinesin motor domains, there exists a high degree of conservation, both structurally and mechanistically, both within and between species. Briefly, the enzymatic cycle of a kinesin motor consists of ATP hydrolysis via the switch I and II motifs allowing for changes in microtubule affinity allowing for sequential engagement/re‐engagement with the substrate.^3^ Given the of conservation in the mechanism of kinesin motor domains, it is unsurprising that differences in kinesin motility and direction are due to domains outside the basic motor catalytic unit.

The Kinesin‐3 superfamily of motors is one such group whereas the physical properties of the motor have been extensively studied. The Kinesin‐3 superfamily consists of the KIF1, KIF13, KIF14, KIF16, and KIF28 subfamilies.^4^ Additionally, Kinesin‐3 motors are highly conserved across species.^4^, ^5^ Structurally, motor domain Loop 12 of Kinesin‐3 motors contains a string of lysine residues that function to enhance the microtubule on‐rate of these motors in their ADP bound state and the enhanced on‐rate is due to an electrostatic interaction between the K‐loop and the glutamate‐rich E hook found on the exposed C‐terminal tails of beta‐tubulin subunits in polarized microtubules.^6^ Additionally, Kinesin‐3 motors contain conserved neck‐coil (NC) and at least one coiled‐coil domain (CC) immediately adjacent to their motor domains. The NC‐CC domains function to maintain these motors in autoinhibited states until cargo binds.^7^ Upon cargo binding, the mechanistic properties of the NC‐CC domains function to greatly enhance the ability for motors in the Kinesin‐3 group to traverse long distances along microtubule substrates.^8^ When combined, increased on‐rates and long run‐lengths both contribute to the high processivity of Kinesin‐3 motors. Not surprisingly, Kinesin‐3 motors are enriched in cells requiring long distance trafficking, such as neurons. In fact, the founding member of the kinesin‐3 family, UNC‐104 (KIF1A in mammals), was identified due to the severe locomotive defects associated with lack of synaptic vesicle delivery in the ventral nerve cord (VNC) neurons of *Caenorhabditis elegans*.^9^ Further identification of the various Kinesin‐3 motors and cargoes has begun to shed light on the varied roles of these proteins, especially in neurons; both in development and in higher order neurological behaviors. In mice, KIF13A is at least partially responsible for trafficking of the 5HT1A receptor with, knockout of the motor resulting in increased anxiety, but no other developmental defects.^10^ Likewise, a mutation in the cargo binding domain of KIF1Bß significantly alters the trafficking of IFG1R in neurons, contributing to reduced axonal growth and potentially exacerbating phenotypes associated with Charcot‐Marie‐Tooth disease.^11^

The mechanism of Kinesin‐3 motor processivity has been extensively documented through elegant use of both in vitro and ex vivo methods, raising questions as to how these highly processive motors are utilized in vivo. The nematode *C elegans* provides an excellent system to answer these questions, especially considering the extensive work done in the model identifying neuronal wiring.^12^ There are at least three members of the Kinesin‐3 superfamily have been identified in *C elegans* via sequencing homology with their mammalian counterparts; *unc‐104*/KIF1A*, klp‐6, and klp‐4*/KIF13A/KIF13B.^5^ As with other Kinesins, the nematode Kinesin‐3 motors show high conservation in their N‐terminal motors, stalks, and rods, and diverge significantly in their C‐terminal regions presumably to discriminate between different and unique cargoes. Motor‐dependent transport of synaptic cargoes is of particular interest, since this mechanism can provide robust and timely transport relative to lateral diffusion. As mentioned above, UNC‐104 is extensively studied, especially for its roles in axonal trafficking.^9^, ^13^ Detailed genetic analysis has established roles for axonal access, motor activation, active zone components, and synaptic retention.^14^, ^15^, ^16^, ^17^, ^18^ KLP‐6 on the other hand, shows a slightly different expression pattern in worms, being predominantly expressed in heard neurons. The function of KLP‐6 has been closely tied to head ciliated sensory neurons, where KLP‐6 is integral to the release of ciliary vesicles into the environment.^19^ KLP‐4 is the least well understood Kinesin‐3 in *C elegans*. Previous work on this motor in the context of trafficking the glutamate receptor, GLR‐1, in neurons, where *klp‐4* loss of function alleles conferred glutamatergic signaling defects.^17^, ^20^

Here we sought out to further describe the in vivo role of KLP‐4. To this end, we characterized the effect of a previously generated allele, *klp‐4(ok3537)*, on the animal's neuronal development and behavior. We determined that *ok3537* represents a likely indel of *klp‐4*. This *klp‐4* allele appears to be expressed, allowing us to assess some of the motor's mechanism and function in the context of the entire living organism. Whereas *unc‐104* mutants have severe uncoordinated phenotypes, *klp‐4* mutant animals display less severe locomotive defects, however, these defects do include those associated with altered cholinergic signaling. Furthermore, KLP‐4 is involved in establishing proper organization of synaptic vesicles (SVs) in VNC axons. Additionally, locomotive phenotypes can be rescued by overexpression of the wild type motor. These data establish a general role of KLP‐4 and gives mechanistic insight into how Kinesin‐3 motors function in vivo.

## 2 MATERIALS AND METHODS

### 2.1 Worm strains and husbandry

All strains were maintained as outlined by Brenner.^21^ A notable addition to standard husbandry protocol was the addition of nystatin (Millipore Sigma, Darmstadt, Germany) to a final concentration of 10 µg/mL in normal growth media (NGM) plates to control fungal growth. Animals were passaged to fresh plates every 4‐5 days. Strains were obtained from the *Caenorhabditis* Genetics Center (CGC, University of Minnesota‐Twin Cities, St. Paul MN), funded by the NIH Office of Research Infrastructure Programs (P40 OD010440). A list of strains utilized in this study can be found in Supplementary Table S1.

### 2.2 PCR/RT‐PCR/Sequencing

All primers were purchased from Integrated DNA Technologies (IDT, Coralville, IA). For end point reverse transcription PCR analysis, total mRNA was isolated from 10 young adult animals using Trizol (Millipore Sigma) followed by ethanol precipitation. Samples were DNase (New England Biolabs, NEB, Ipswich, MA) treated to remove genomic DNA contamination and the subject to amplification with the OneTaq One Step RT PCR kit (NEB) per manufacturers protocol. For genomic DNA PCRs, single young adult worms were picked in lysed in worm lysis PCR buffer (10 mmol/L Tris, 50 mmol/L KCl, 1.5 mmol/L MgCl_2_, pH 8.3 with 1 mg/mL Proteinase K (NEB)). One μL of genomic DNA lysate was used in PCR reactions using OneTaq DNA polymerase (NEB). Semi‐quantitative end point RT analyses were run in triplicate and mRNA isolation was done as above. Images were captured on a LI‐COR Odyssey FC Imaging System (LI‐COR Biosciences, Lincoln, NE) and analyzed using LI‐COR Imaging Studio Lite. Samples were normalized to actin from the identical sample during quantification. All sequencing was performed by Eurofins Genomics (Eurofins Americas, Louisville, KY).

### 2.3 Behavior assays

Spontaneous reversal frequency was assayed by moving single worms to fresh NGM plates and allowing them to equilibrate for at least 10 minutes. Single worms were recorded for up to 10 minutes using a Motic SMZ‐171‐TLED stereomicroscope (Motic North America, Richmond, BC, Canada) equipped with a MoticamX digital camera and counting the number of times an individual worm reversed per minute. A reversal was characterized as any retrograde movement that exceeded the length of the worm's pharynx. Response to nose touch was assayed by placing animals on fresh NGM plates, allowing them to equilibrate, and then challenging the animal with a hair on the agar surface so that the worm contacted the hair with its nose at a 90‐degree angle. In this assay, normal worms were characterized by immediate reversal upon contact with the obstacle. Defective animal behavior was characterized as any behavior that deviated from the normal behavior, such as the worm moving its nose along the hair or attempting to hurdle over the obstacle. Each worm was tested no more than 10 times. Osmotic avoidance was assayed by washing animals extensively with M9 buffer (22 mmol/L KH_2_P0_4_, 42 mmol/L Na_2_HPO_4_, 86 mmol/L NaCl, all chemicals from Millipore Sigma) and the placing animals inside a ring of 8M glycerol‐trypan blue (both from Millipore Sigma) solution spotted on an NGM plate without food. After 10 minutes, the number of animals escaping the ring were counted and compared to the total number of animals in the assay.

### 2.4 Locomotive assays

Aldicarb assays were run as described in Oh and Kim.^22^ Briefly, aldicarb (Millipore ‐Sigma) stock was added to agar plates to a final concentration of 0.5 mmol/L during the plate pouring process. Worms were transferred to each plate and the time course of paralysis was monitored in 30‐minute intervals over the next 3 hours. Paralysis was indicated by a failure of the animal to move when prodded with a platinum wire. For transgenic rescue experiments, animals positive for the transgenic co‐injection marker were subject to an aldicarb sensitivity assay and compared to non‐transgenic siblings from the same plate. For serotonin hypersensitivity assays 200 µL of serotonin solution (40 mmol/L serotonin creatinine sulfate monohydrate (Millipore Sigma) in M9) were added to a 96 well plate. Twenty worms were transferred to each well and percentage of worms paralyzed following a five‐minute time‐period was recorded.

### 2.5 Statistical analysis

Welch's ANOVA and Games‐Howell post hoc analysis were completed using an online tool found at (http://www.biostathandbook.com/onewayanova.html). ANOVA and Tukey‐Kramer HSD tests were completed using an online tool found at the following URL: (http://astatsa.com/OneWay_Anova_with_TukeyHSD/) and graphs constructed using Excel and Adobe Illustrator.

### 2.6 Confocal microscopy

All images were captured using a Zeiss ISM 700 Laser Scanning Confocal Microscope (Carl Zeiss Microscopy LLC, Thornwood, NY). VNC pictures were taken immediately posterior to the vulva. For maximum intensity projections, a z‐series of images was collected and collapsed into a single image. Collected images were processed using Adobe Photoshop.

### 2.7 Transgenesis

Production of transgenic strains were created as described by Mello.^23^ For *klp‐4* rescues, the fosmid UBC_f80C1340Q was obtained from The BioSource Project (Nottingham UK). The strain RB2546 containing the *klp‐4(ok3537)* allele was injected with 50 ng/µL rescue fosmid and 30 ng/µL of P*unc‐122*::*unc‐122*::eGFP co‐injection marker. F1 progeny positive for the co‐injection marker was cloned to generate independent transgenic lines.

## 3 RESULTS

### 3.1 *ok3537* is an in‐frame deletion in the cargo binding domain of *klp‐4*

**Figure 1:**
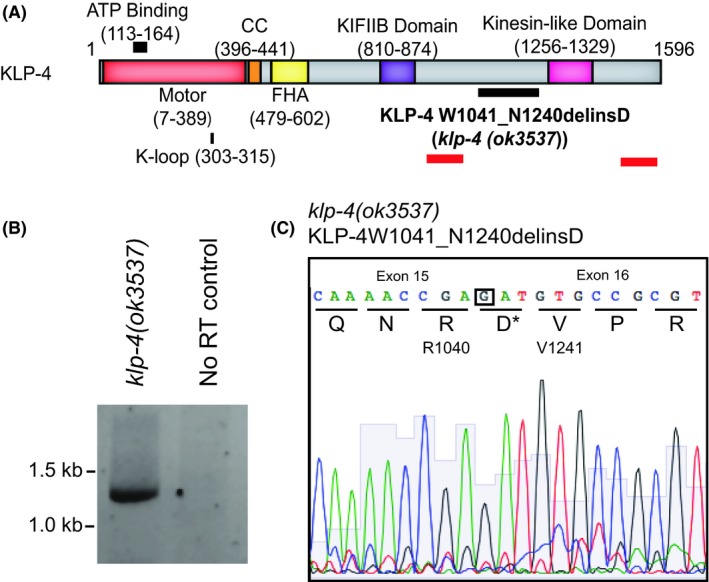
Identification of the *klp‐4(ok3537)* indel allele. (A) Schematic of the *klp‐4* gene. The location of the *ok3537* indel is marked by a solid black line. Location of primers used in subsequent sequencing analysis marked by solid red lines. (B) RT‐PCR analysis of *klp‐4* DNAs. cDNAs were reverse transcribed from *klp‐4(ok3537)* mutants. Expected size of *klp‐4(ok3537)* cDNA in this reaction is 1210 bp. (C) Chromatogram of *klp‐4(ok3537)* cDNA sequence. The translation is indicated below nucleotide base calls. The insertion of a single G maintains the reading frame of the predicted protein. Insertion of a single aspartic acid (D) is marked with an *

To analyze the role of the Kinesin‐3 motor, KLP‐4 in vivo*,* we obtained a commercially available strain containing a minimally characterized allele, *klp‐4(ok3537)* (Figure 1A). The *klp‐4(ok3537)* allele contains a large deletion in the predicted cargo binding domain of the protein; however, the exact position of the mutation remained unknown. To determine the exact nature of this allele, we isolated cDNA from the strain RB2546 which contains the *klp‐4(ok3537)* mutation and amplified the sequence surrounding the mutation (Figure 1A). This mutant allele deletes 647 nucleotides from the gene and inserts a single guanosine at this location. This large deletion removes a portion of exon 15, the entirety of intron 15, and a 5’ portion of exon 16, however, the insertion of a single G allows the predicted reading frame to be maintained, resulting in the indel mutation W1041_N1240delinsD. To test for expression, cDNA was produced from *klp‐4(ok3537)* containing animals. The results of an PCR from *klp‐4(ok3537)* cDNA produced a DNA band of 1210 bp, which is the predicted product size of a PCR from cDNA containing this deletion with the primer sets utilized (Figure 1B). Sequencing of cDNA from this PCR reaction confirmed that we were able to isolate mRNA from these animals containing this mutation, suggesting this transcript is not destroyed by nonsense mediated decay in *klp‐4(ok3537)* mutant animals (Figure 1C).

### 3.2 *klp‐4* mutants have altered locomotive behavior

**Figure 2:**
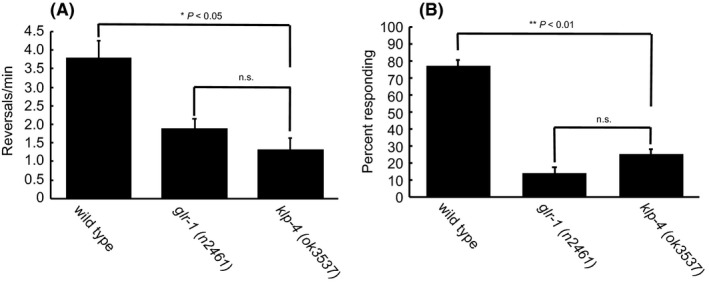
*klp‐4(ok3537)* mutants have locomotive defects. (A). Spontaneous reversals per minute of wild type (n = 30), *glr‐1(n2461)* (n = 30) controls, and *klp‐4(ok3537)* (n = 20) mutant animals. Error bars are SEM. P‐values indicated for significant differences (one‐way Welch's ANOVA with Games‐Howell post hoc test). (B) Nose touch response assay. Wild type, *glr‐1(n2461),* and *klp‐4(ok3537)* animals were challenge by perpendicular nose touch no more than 10 times. n = 15 animals per genotype. Error bars are SEM. *P* values indicated for significant differences (one‐way ANOVA with Tukey HSD test)

KLP‐4, like other Kinesin‐3 motors, is highly expressed in neurons.^24^ We wanted to determine what role, if any KLP‐4 might have in neurons considering that other Kinesin‐3 motors are critical to the animals’ behaviors.^9^, ^19^ Monitoring of spontaneous reversals can be used to assess behavioral changes to stimuli.^25^ Spontaneous reversals monitoring has also previously been utilized to assess behavior defects other alleles of *klp‐4* due to the role of glutaminergic signaling in this process.^17^, ^20^ *klp‐4(ok3537)* mutants display significantly fewer spontaneous reversals than wild‐type worms, and similar numbers of reversals relative to *glr‐1* mutant controls (Figure 2A). These results suggest the *ok3537* allele also leads to general neuronal defects in vivo.

The *glr‐1* associated phenotypes associated with *klp‐4* mutants prompted us to investigate if further locomotive defects were present in these animals. Kinesin‐3 motors, such as *unc‐104*/KIF1A are essential for locomotive behavior and since *klp‐4* is expressed in the same subset of locomotive neurons as *unc‐104*, we next assayed whether *klp‐4(ok3537)* animals also display defects in locomotion. Considering that *klp‐4* mutants do not display obvious uncoordinated (Unc) phenotypes, we assayed locomotion using nose‐touch challenge, where animals should be able to recognize a challenge and initiate the proper evasive response signaling clear cross talk between forward and reverse locomotive circuits utilizing VNC command interneurons.^26^, ^27^ In response to nose touch, *klp‐4(ok3537)* worms failed to properly respond to challenge as compared to wild‐type animals, however, *klp‐4* mutants did responds similar to nose touch defective *glr‐1* animals (Figure 2B). The results of these nose touch assays suggest a role for *klp‐4* in locomotive neuronal signaling and implicate that the VNC interneurons comprising the locomotive circuitry are affected.

### 3.3 *klp‐4* mutants have defects in cholinergic signaling

**Figure 3:**
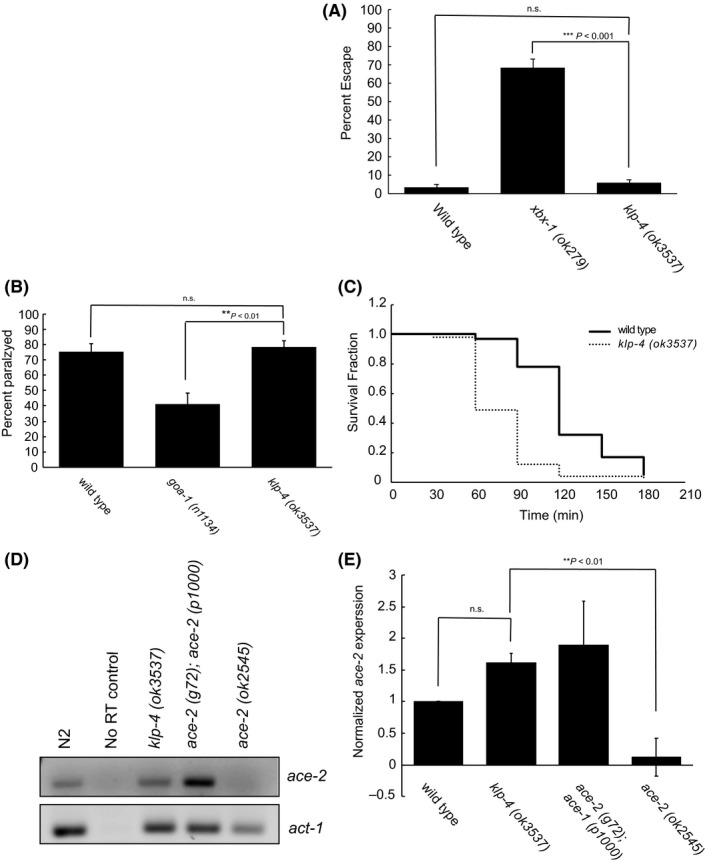
*klp‐4(ok3537)* mutants have altered cholinergic signaling. (A) Osmotic avoidance assay. Animals were challenged to escape an 8M glycerol ring in 10 minutes, and mean percent escaping was calculated. Wild type n = 11 trials, *xbx‐1(ok279)* n = 7 trials, *klp‐4(ok3537)* n = 9 trials. Error bars are SEM. *P*‐values indicated for significant differences (Welch's ANOVA with Games‐Howell post hoc test). (B) Serotonin immobilization assay. Animals were treated with 40 mmol/L exogenous serotonin and the percent paralyzed after counted after 5 minutes. Wild type n = 8 trials *goa‐1(n1134)* n = 7 trials, *klp‐4(ok3537)* n = 10 trials. Minimum 20 animals per trial. Error bars are SEM. *P*‐values indicated for significant differences (Welch's ANOVA with Games‐Howell post hoc test). (C) Compiled aldicarb sensitivity assay. Animals were incubated on plates containing 0.5 mmol/L aldicarb and assayed for paralysis every 30 minutes for 180 minutes. Kaplan‐Meier plot with fraction surviving for each time point are indicated. *P*‐values for Log‐rank tests for each trial and compiled data can be found in Table 1. Wild type: solid line, *klp‐4(ok3537)*: dashed line. Data representative of five independent trials, minimum 20 animals per trial. (D) Semi‐quantitative end point reverse transcription PCR of *ace‐2. ace‐2* functional null (*ace‐2(g72))* and genetic null (*ace‐2(ok2545)*) alleles were used as controls. Actin (*act‐*1) was used as a loading control and to normalize data. (E) Quantification of results shown in (D). Reactions were run in triplicate triplicates. Asterisk denotes significance in a one‐way ANOVA with Tukey HSD test. Error bars represent SEM

We next asked which signaling pathways might be disturbed due to the above behavioral defects observed. To assess the signaling pathways associated with the above phenotypes, we subjected *klp‐4* mutant animals to different assays designed to isolate specific signaling pathways. Since homologs of *klp‐4* have also been associated with ciliary signaling,^19^, ^28^ we tested *klp‐4* mutants for ciliary signaling defects. *C elegans* utilizes both kinesin motors localized to the ciliary sensory neurons to actively avoid high areas of high osmotic strength.^29^ If *klp‐4* functions in ciliated sensory neurons, then we might observe an inability to sense highly concentrated solutions. However, when assayed for osmotic avoidance defects but found no significant differences relative to wild type animals demonstrating that *klp‐4* is not required for osmolarity sensing (Figure 3A).

Serotonin signaling is also associated with locomotive behaviors. When worms are treated with exogenous serotonin, their locomotion significantly decreases.^30^ Additionally, KIF13A conditional knockout mice have 5HT receptor trafficking defects resulting in anxiety as measured by an inability to perform certain behaviors.^10^ These links prompted us to ask if serotonin signaling was at the root of *klp‐4* mutant locomotive defects. Therefore, we asked if *klp‐4* mutant animals might be more or less sensitive to exogenous serotonin. As a control, we included *goa‐1* mutant animals known to be less sensitive to the effects of serotonin. Using a serotonin immobilization assay, we found no difference between *klp‐4* mutants and wild type animals, suggesting serotonin signaling is unaffected here (Figure 3B).

Command interneurons found in the VNC are cholinergic,^12^ we also asked if cholinergic signaling was disrupted in *klp‐4(ok3547)* animals. Aldicarb is a potent acetylcholinesterase inhibitor and has been traditionally used to assess this signaling pathway by blocking the breakdown of acetylcholine.^31^ Animals with normal cholinergic signaling will eventually become paralyzed in the presence of aldicarb. When plated on NGM plates containing 0.5 mmol/L aldicarb, *klp‐4* mutants were found to be hypersensitive and were paralyzed nearly 50% faster than wild type animals (Figure 3C, Table 1). To eliminate the possibility that this aldicarb hypersensitivity phenotype seen in *klp‐4* mutants is due to specifically to the mutation in question and not due to reduced levels of acetylcholinesterase (AchE), we also analyzed expression of the AchE gene, *ace‐2. ace‐2* is the major acetylcholinesterase found in VNC neurons.^32^ Using semi‐quantitative RT‐PCR and densitometry, we found no significant differences in the expression of *ace‐2* between *klp‐4* mutants and wild type worms (Figure 3D and 3). As controls, we also included both *ace‐2* functional null animals and *ace‐2* genetic nulls. These data demonstrate that *klp‐4* specifically effects locomotion based on defects in cholinergic signaling, and the cholinergic phenotypes displayed likely result from and increased level of acetylcholine at the synapses of the locomotive circuitry.

**Table 1:** Median survival time (minutes) of *Caenorhabditis elegans* exposed to 0.5 mmol/L aldicarb

	Trial 1	Trial 2	Trial 3	Trial 4	Trial 5	Compiled
Wild type	120 (n = 20)	120 (n = 20)	120 (n = 20)	150 (n = 20)	120 (n = 20)	120 (n = 100)
*klp‐4 (ok3537)*	60 (n = 20)	60 (n = 20)	60 (n = 20)	90 (n = 20)	60 (n = 20)	60 (n = 100)
*P*‐value	<0.0001	<0.0001	<0.01	<0.01	<0.001	<0.0001

*P*‐values for Log‐rank test indicated for each trial and compiled data.

### 3.4 Organization of synapses is KLP‐4 dependent

**Figure 4:**
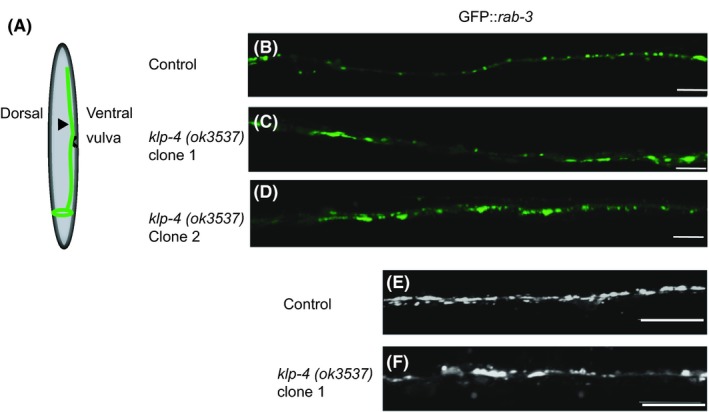
*klp‐4(ok3537)* mutation leads to disorganization of ventral nerve cord (VNC) axons. (A) Schematic of worm imaging. Black arrowhead indicates approximate location of all representative images. (B‐D) Confocal images of an integrated *GFP::rab‐3* transgene in VNC axons, in (B) control and (C and D) *klp‐4(ok3537)* mutant backgrounds. (E and F) Zoom of maximum intensity projections of collapsed z‐stacks of VNCs in control and *klp‐4(ok3537)* mutant backgrounds. All images taken just posterior to the vulva in age‐matched young adult hermaphrodites. All scale bars 10 μm

The AVB interneuron found in the VNC of *C elegans* is the major command interneuron responsible for integrating forward locomotive behavior.^27^ Using a GFP transcriptional reporter for the AVB interneuron, we did not identify any gross morphological or developmental defects in AVB in *klp‐4(ok3537)* mutants signifying that any locomotive defects in these animals are most likely due to incorrect distribution of subcellular components (data not shown). To assess the subcellular effects of *klp‐4* mutation, we used a GFP::RAB‐3 reporter to visualize the VNC. When we cross our *klp‐4(ok3537)* mutants with a GFP::RAB‐3 stain we observed that the VNC of mutant worms appear disorganized and that RAB‐3 positive structures were aggregated compared to controls (Figure 4B‐D). Additionally, *klp‐4* mutants had ectopic blebbing and general disorganization of RAB‐3 positive SVs in VNC interneurons (Figure 4E and F). These phenotypes were highly penetrant in *klp‐4(ok3537)* mutants. Taken together, these results suggest that the *klp‐4(ok3537)* mutation specifically results in altered locomotive behavior based on the inability to correctly organize the VNC.

### 3.5 Aldicarb hypersensitivity can be rescued by overexpression of KLP‐4

**Figure 5:**
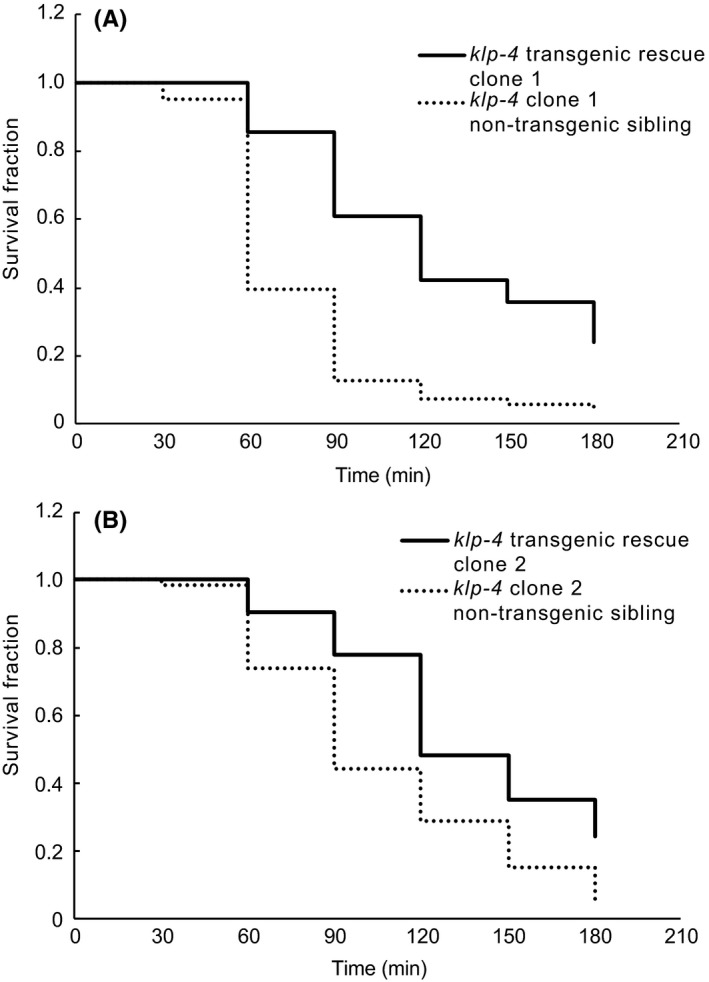
*klp‐4(ok3537)* cholinergic hypersensitivity can be rescued by wild type *klp‐4* overexpression. Aldicarb assays of two independent rescue clones (A, clone 1, B clone 2) compared to non‐transgenic sibling controls. Animals were incubated on plates containing 0.5 mmol/L aldicarb and assayed for paralysis every 30 minutes for 180 minutes. Kaplan‐Meier plot with fraction surviving for each time point are indicated. *P*‐values for Log‐rank tests for each trial and compiled data can be found in Table 2. *klp‐4(ok3537)* rescue clones: solid line, non‐transgenic sibling control: dashed line. Data representative of five independent trials for clone 1 and 6 independent trials for clone 2

Cholinergic signaling in *C elegans* is extensively regulated including at the levels of transcription, developmental time, and sexual dimorphism.^12^ Also, expression mutant kinesins can lead to a dominant negative effect by potentially dimerizing with wild type counter parts.^33^ Therefore, in order to assess whether the *klp‐4(ok3537)* mutation was solely responsible for our aldicarb hypersensitivity phenotype, we used a fosmid containing the wild type copy of *klp‐4* in rescue experiments. The fosmid used in these experiments contains the endogenous *klp‐4* promoter region, ensuring that *klp‐4* expression is controlled by its endogenous transcriptional regulators. Likewise, by utilizing fosmid based overexpression, we can potentially eliminate any dominant negative effects. We found that *klp‐4* aldicarb hypersensitivity can be rescued by introduction of the wild type sequence of *klp‐4* as compared to non‐transgenic siblings of the same independent clone (Figures 5A and 5B, Table 2), suggesting that cholinergic signaling phenotypes are specific for defects in *klp‐4*.

**Table 2:** Median survival time (minutes) of *klp‐4(ok3537)* animals +/− a *klp‐4(+)* rescue transgene during exposure to 0.5 mmol/L aldicarb

	Trial 1	Trial 2	Trial 3	Trial 4	Trial 5	Trial 6	Compiled
*klp‐4* transgenic rescue clone 1	90 (n = 14)	90 (n = 20)	90 (n = 41)	120 (n = 44)	140 (n = 45)	120 (n = 16)	120 (n = 180)
*klp‐4* clone 1 non‐transgenic sibling	90 (n = 38)	60 (n = 26)	60 (n = 82)	90 (n = 12)	60 (n = 15)	90 (n = 17)	60 (n = 190)
P‐value	ns	<0.05	<0.05	<0.0001	<0.0001	<0.05	<0.0001
*klp‐4* transgenic rescue clone 2	120 (n = 41)	120 (n = 27)	120 (n = 25)	180 (n = 16)	150 (n = 19)	n/a	120 (n = 128)
*klp‐4* clone 2 non‐transgenic sibling	90 (n = 40)	90 (n = 22)	90 (n = 41)	120 (n = 28)	120 (n = 26)	n/a	90 (n = 157)
*P*‐value	<0.01	ns	<0.05	<0.05	<0.05	n/a	<0.0001

*P*‐values for Log rank test indicated for each trial and compiled data.

## 4 DISCUSSION

In this study we describe an in‐frame deletion allele for the *C elegans* Kinesin‐3 motor, KLP‐4. Subsequent behavior and imaging analyses highly suggest that this in‐frame deletion leads to an expressed, yet shortened motor. Using this mutant *klp‐4* allele, we were able to uncover KLP‐4 contributes to the cholinergic based locomotive circuitry of *C elegans*. Defects in Kinesin‐3 motor function have been implicated in numerous neurological defects in higher eukaryotes,^34^ necessitating an understanding of how these motors function in vivo. Classically, the nematode has been used as model for kinesin biology, with UNC‐104/KIF1A being the prototypical motor responsible for anterograde trafficking of cargoes along axons, including the VNC cholinergic interneurons.^9^ Cargoes of UNC‐104 include SVs and active zone proteins required for maintenance of synaptic density and size.^18^ The related kinesin, KLP‐4 (KIF13A and KIF13B in humans) has a similar cellular distribution pattern to UNC‐104^35^ prompting us to inquire about the function of this motor in the same subset of VNC neurons.

Questions remain as to the nature of Kinesin‐3 motors; in terms of their active versus inactive states and how this transition occurs. Current in vitro and cell‐based mammalian models of Kinesin‐3 motors, including the KLP‐4 homologs, suggest motor activation requires cargo binding to induce dimerization, with intramolecular binding between NC and CC regions maintaining the motor as an inactive monomer until cargo binding.^8^ Structural data supports this model, with the notable additions of CC domain and NC domains binding onto the motor domain (MD) preventing neck linker (NL) undocking from the MD and release of ADP from the MD, therefore inhibiting dimerization.^7^ Motor domain autoinhibition by the CC domains is common in other Kinesin‐3 motors including UNC‐104/KIF1A.^18^, ^36^ The *klp‐4(ok3537)* allele utilized in this study does not disrupt either the NC or CC domains of the motor, suggesting that these motors should be able to maintain their autoinhibited, monomeric state until, presumably, cargo is bound. It is tempting to speculate that a significant in frame deletion in the CBD of the motor would result in constitutive activation since CBD inhibition of MDs is seen in other kinesins.^33^ MD specific autoinhibition by the CBD has been observed for mammalian KIF13B, working in conjunction with Par1b kinase and 14‐3‐3β protein binding.^37^ However, blocking autoinhibition of KIF13B leads to excessive axonal protrusions in culture neurites, a phenotype we did not observe here.^37^ When combined with both structural data and the location of the deletion for the allele in this study, evidence argues strongly against CBD autoinhibition in KLP‐4. The small GTPase, ARL‐8, is required to active UNC‐104/KIF1A via alleviation of CC1‐CC2 autoinhibition status.^18^ Given the high conservation of Kinesin‐3 motors, it is likely that KLP‐4 also contains an “activator” domain required to maintain the motor as a compact monomer. CDK‐5 one such candidate for regulating KLP‐4 in vivo. CDK‐5 and KLP‐4 function in the same genetic pathway and double loss of function mutants for each gene result in cargo accumulation and degradation in the soma.^20^ Additionally, CDK‐5 also works in the same genetic pathway as another neuronal kinesin‐3, UNC‐104,^13^ therefore these motors might work in the same axonal entry pathway or that they have overlapping functions in neurons.

Cargo binding is essential to Kinesin‐3 activation therefore genetic analysis of different *klp‐4* alleles and the impact these alleles impact their cargoswill give mechanistic insight has to how Kinesin‐3 motors function in vivo. Reverse transcription analysis and sequencing showed that despite a the large indel found in *klp‐4(ok3537)*, the reading frame is maintained, resulting in a protein with a predicted 200 amino acid deletion in its CBD (Figure 1). With a deletion in only of part of the CBD, this version of KLP‐4 might be able to bind some, but not all of its cargoes. There exist two other alleles for *klp‐4* that can be used for comparison to determine the functionality of this motor and its role in axonal trafficking. One such allele, *klp‐4(pz19)* contains a premature stop codon in the CBD, whereas *klp‐4(tm2114)* is a significant deletion in the MD coding region. Both these *klp‐4* alleles show decreased axonal localization of the the glutamate receptor, GLR‐1 as well as the associated *glr‐1* deficient behaviors.^20^ We were able to recapitulate similar *glr‐1* behavioral phenotypes (Figure 2) as previously described.^20^ Importantly however, these *klp‐4* null mutants do not display altered trafficking of numerous synaptic markers whereas the *klp‐4(ok3537)* shows an increase of a synaptic marker in the VNC.^20^ Here we observe the opposite; an accumulation of cargo in the axon (Figure 4) suggesting that the *klp‐4(ok3537)* allele might represent at least a somewhat functional motor. Furthermore, analysis of GLR‐1 containing vesicles in a *klp‐4(ok3537)* background shows decreased size of GLR‐1 containing puncta, but GLR‐1::GFP containing vesicles maintain both run length and velocity, as well as signal intensity clearly establishing a role for KLP‐4 at some point in the axonal trafficking pathway.^17^ These authors suggest that KLP‐4 does not participate in long range trafficking of GLR‐1 in axons, yet effects cargos in the soma to regulate the amount of cargo entering the axon.^17^ Given that the *klp‐4(ok3537)* mutation only removes some, but not all of the motor's CBD, it seems plausible that it could represent a motor with the ability to dimerize and become active, but due to the CBD deletion can only exert effects on certain cargos, like GLR‐1 not others such as RAB‐3. Although our data cannot directly confirm the functionality of the motor, a partial null or constitutively active Kinesin‐3 would prove to be valuable in assessing Kinesin‐3 cargo binding and regulatory mechanisms in vivo and are the focus of ongoing investigation.

Additional cargos have been identified for mammalian homologs in other models that might also give insight to both our behavior and structural phenotypes. Domain specific analysis of KIF13B in mammals has demonstrated that KIF13B likely interacts with two important polarity determining factors; the Discs large (Dlg) tumor suppressor and the PIP3 adapter protein centaurin‐alpha 1.^38^, ^39^, ^40^, ^41^, ^42^ Binding sites for both family of proteins are near the motor's N‐terminal FHA domains. *klp‐4(ok3537)* animals do not demonstrate any gross morphological defects in development of VNC interneurons suggesting trafficking of proteins, like Dlg, or PIP3 vesicles are unlikely impaired, which is not surprising since *ok3537* maintains the motors FHA domain and the Dlg binding domain resides. In mammals, mannose‐6 phosphate receptor (M6PR) is transported by KIF13A via the AP‐1 adaptor binding to the motor's CBD,^43^ although *C elegans* lack a true homolog of the M6PR it is important to note that AP‐1 and the related AP‐3 adapter proteins are required for post‐Golgi sorting of dendritic and axonal cargoes respectively.^44^ Taken together, these data suggest that *klp‐4(ok3537)* codes for a motor localized to the soma that is capable of binding some cargoes required for proper neuronal development but cannot bind other cargoes required for proper axonal sorting.

The small GTPase RAB‐3 has been historically used as a marker of synaptic vesicles in multiple organisms, including the nematode. In *unc‐104* loss of function mutants, RAB‐3 accumulates in cell bodies.^31^ *unc‐104(gf)* mutation results in a decrease in synaptic density in axons and produces resistance to aldicarb.^18^ Here, one of the subcellular phenotypes associated with *klp‐4(ok3537)* is a disorganization SVs marked by GFP::RAB‐3 localization in axons (Figure 4). Cholinergic signaling is the primary neurotransmitter involved with worm locomotion, and RAB‐3 positive SVs are presumably responsible for carrying acetylcholine production machinery; the acetyltransferase *cha‐1* and the vesicular acetylcholine transporter *unc‐17*.^31^, ^45^ We hypothesized that given the overabundance and disorganization of RAB‐3 axonal vesicles, that cholinergic signaling should be disrupted in *ok3537* animals. These animals are hypersensitive to aldicarb as predicted, and this phenotype can be rescued with a *klp‐4* wild type transgene (Figures 4 and 5). These data demonstrate a role for *klp‐4* in cholinergic signaling and will help to further characterize the mechanism of acetylcholine release and signaling network regulation in vivo.

Previous studies have demonstrated similar axonal organizational phenotypes as we see in our *klp‐4* mutant animals. It is interesting to note that our GFP::RAB‐3 phenotype appears to extend the length of VNC axons. The motor, KIF5/UNC‐116 traffics GLR‐1 in the AVA neuron of the VNC and strong reduced function of *unc‐116* in the VNC leads to aggregation of GLR‐1::GFP.^17^, ^46^ This organizational phenotype in *unc‐116* mutants is restricted to the proximal axon. ARL‐8 is a regulator of SV aggregation, and as mentioned previously activates the related UNC‐104 kinesin.^47^ RAB‐3 positive SV clustering in *arl‐8* mutants was also restricted to the proximal region of the axon similar to *unc‐116* mutants and did not extend the length of the structure as observed in this study, suggesting that KLP‐4 is part of a more general axonal delivery and/or removal pathway.

Intensive genetic analyses provide a number of candidate proteins that might function with KLP‐4 to regulate axonal entry and/or accumulation of cargoes. The highly conserved core synapse stability (CSS) system, consisting of Sentryn, SAD‐1*,* SYD‐1*,* and SYD‐2*,* plays intricate roles in synaptic vesicle guided transport from soma to synapse and capture of SVs and dense core vesicles (DCVs) at active zones in axons.^14^ CSS mutants for *syd‐1, syd‐2,* and *sad‐1* antagonize *arl‐8* mutations.^47^ The CSS system regulates UNC‐104/KIF1A anchoring of SVs at synapses,^16^ leaving the possibility that KLP‐4 might also be regulated by or be part of the same cellular constituents regulating the axonal guided transport process. Also, UNC‐16/JIP3/Sunday Driver is as a gatekeeper for axonal access,^16^, ^48^ acts in early sorting/vesicular biogenesis events,^49^ and functions upstream of the CSS system.^16^ in the absence of UNC‐16, synaptic markers are aggregated in axons, therefore it seems plausible that UNC‐16 also regulates KLP‐4 thus contributing to axonal targeting of RAB‐3 vesicles as part of the UNC‐16 regulated pathway. It should also be noted that the previously mentioned CDK‐5 works downstream of UNC‐16 in SV trafficking^16^ and CDK‐5 works in the same genetic pathway as KLP‐4.^20^ Therefore, KLP‐4 could be working as part of a concerted pathway to restrict cargoes from entering the axon, and changes in KLP‐4 functionality could allow certain cargoes, such as RAB‐3 positive vesicles, enfettered access to the axon.

## CONFLICT OF INTEREST

The authors declare no conflicts of interest concerning this submission.

## AUTHOR CONTRIBUTIONS

MM assisted with the design of the study and performed experiments. KP performed experiments and produced reagents. CD performed experiments and produced reagents. JP designed study, provided guidance, performed experiments, prepared reagents and prepared this manuscript.
