# Supplementary material for: The Kinesin‐3 motor, KLP‐4, mediates axonal organization and cholinergic signaling in Caenorhabditis elegans
Source: FASEB Bioadv. 2019 Jun 11;1(7):450–60. doi: 10.1096/fba.2019-00019 (PMC6996341; doi:10.1096/fba.2019-00019)
Supplement: Supplementary file 1 [file FBA2-1-450-s001.pdf]

Supplementary Table 1:

Strains utilized in this study:

**Strain**	**Genotype**	**Source**
N2	wild type	CGC
RB2546	*klp-4(ok3537)*	CGC
KP4	*glr-1(n2461)*	CGC
MT2426	*goa-1(n1134)*	CGC
JT11069	*xbx-1(ok279)*	CGC
GG201	*ace-2(g72); ace-1(p1000)*	CGC
RB1942	*ace-2(ok2545)*	CGC
NM2415	*prab-3::gfp::rab-3*	CGC
PVX38	*klp-4(ok3537);prab-3::gfp::rab-3*	Cross NM2415 x RB2546
PVX39	*klp-4(ok3537);prab-3::gfp::rab-3*	Cross NM2415 x RB2546
PVX60	*klp-4(ok3537);mvpEx4(punc-122::unc-122::egfp; klp-4(+))*	Transgenesis of RB2546 with fosmid clone # UBC_f80C1340Q (The BioSource Project)
PVX62	*klp-4(ok3537);mvpEx4(punc-122::unc-122::egfp; klp-4(+))*	Transgenesis of RB2546 with fosmid clone # UBC_f80C1340Q (The BioSource Project)
